# Targeted Screening with the Use of Clinical Risk Factors for Detecting Congenital Cytomegalovirus Infection in Newborns: A Prospective Multicenter Cohort Study

**DOI:** 10.3390/microorganisms13092197

**Published:** 2025-09-19

**Authors:** Soromon Kataoka, Masatoki Kaneko, Li Yang, Hajime Ota, Moeka Seki, Aya Kobamatsu, Daiki Nakayama, Yu Furuta, Fumie Tanuma, Yoshiyuki Fukushi, Shinichiro Wada, Keiji Haseyama, Hideto Yamada

**Affiliations:** 1 Department of Obstetrics and Gynecology, Hakodate Central General Hospital, Honchou 33-2, Hakodate 040-8585, Hokkaido, Japan; sorokata@hakochu-hp.gr.jp (S.K.); moeka0218mame@gmail.com (M.S.); dnakayama.bz@gmail.com (D.N.); higashinokeigo0511@hotmail.co.jp (Y.F.); tanuma-f@hakochu-hp.gr.jp (F.T.); 2 Department of Obstetrics and Gynecology, Faculty of Medicine, University of Miyazaki, 5200 Kiyotakecho Kihara, Miyazaki 889-1692, Miyazaki, Japan; mkaneko@med.miyazaki-u.ac.jp (M.K.); li_yang@med.miyazaki-u.ac.jp (L.Y.); 3 Department of Obstetrics and Gynecology, Teine Keijinkai Hospital, 1-40, 12-Chome, Maeda, Teine-ku, Sapporo 006-8555, Hokkaido, Japan; hjm.ohta@gmail.com (H.O.); kohta294@yahoo.co.jp (Y.F.); wa_shin_2002@yahoo.co.jp (S.W.); 4 Department of Pediatrics, Teine Keijinkai Hospital, 1-40, 12-Chome, Maeda, Teine-ku, Sapporo 006-8555, Hokkaido, Japan; keiji.haseyama@gmail.com; 5 Center for Recurrent Pregnancy Loss, Teine Keijinkai Hospital, 1-40, 12-Chome, Maeda, Teine-ku, Sapporo 006-8555, Hokkaido, Japan

**Keywords:** congenital infection, cytomegalovirus, screening, newborn

## Abstract

Congenital cytomegalovirus infection (cCMV) is one of the most common congenital infections. This study aimed to evaluate the diagnostic performance of targeted screening with the use of clinical risk factors for cCMV. A total of 3063 pregnant women and their 3139 newborns were enrolled. Six clinical findings consisting of maternal fever or flu-like symptoms during pregnancy (fever/flu-like symptoms), hospitalization for threatened miscarriage or preterm labor before 34 weeks of gestation, preterm delivery before 34 weeks of gestation, fetal ultrasound abnormalities, small for gestational age (SGA), and refer results of automated auditory brainstem response screening (AABR refer) were defined as cCMV risk factors before participant registration. All newborns underwent urine cytomegalovirus polymerase chain reaction tests within one week of birth. The predictive accuracy of these six risk factors was analyzed. Nine (0.29%) of the three thousand one hundred and thirty-nine newborns were diagnosed with cCMV, having at least one of the six risk factors. Logistic regression analysis identified fever/flu-like symptoms (odds ratio (OR), 7.5; 95% CI, 1.9–30.3), fetal ultrasound abnormalities (OR, 17.9; 95% CI, 4.4–72.8), SGA (OR, 6.8; 95% CI, 1.8–25.6), and AABR refer (OR, 75.5; 95% CI, 19.7–289) as significant risk factors. The predictive accuracy of the targeted screening for cCMV, when at least one of the six risk factors was present, yielded 100% sensitivity (95% CI, 55.5–100) and 70.7% specificity (95% CI, 69.1–72.3), with a Youden index of 0.707. When at least one of the four significant risk factors was present, 100% sensitivity (95% CI, 55.5–100) and 81.2% specificity (95% CI, 79.8–82.6) with the maximum Youden index of 0.812 were achieved. In conclusion, targeted screening with the use of clinical risk factors in mothers and their newborns could effectively identify cCMV.

## 1. Introduction

Congenital cytomegalovirus infection (cCMV) is one of the most common congenital infections, with incidence rates ranging from 0.2% to 6.1% [1,2]. Approximately 10–15% of infants with cCMV are born symptomatic, whereas the remaining 85–90% are classified as asymptomatic cCMV [3,4]. Its clinical manifestations include fetal growth restriction, low birth weight, central nervous system findings, multiple organ involvement, petechiae, hepatomegaly, splenomegaly, jaundice, and pneumonia. Among infants with symptomatic cCMV, 40–80% experience long-term neurological sequelae, such as sensorineural hearing loss (SNHL), neuromuscular disorder, psychomotor delay, ocular abnormality, delayed language development, and intellectual disability [5]. In contrast, 10–15% of infants with asymptomatic cCMV exhibit late-onset or progressive neurological impairments, including SNHL and intellectual disability [6,7], and 1.6% of the asymptomatic infants eventually require hearing aids [8].

Both primary and nonprimary infections (reactivation or reinfection with a different CMV strain) can cause cCMV. Approximately 1% of pregnant women with seronegative CMV encounter primary CMV infections during pregnancy, with a vertical transmission rate of 40%. Conversely, in women with seropositive CMV, the vertical transmission rate is 0.5–1%, which is caused by viral reactivation or reinfection [4]. Maternal serological tests for detecting pregnant women with primary CMV infection, including maternal blood tests of CMV-specific immunoglobulins G (CMV IgG) and M (CMV IgM), have been widely used. The gold standard for the diagnosis of primary CMV infection is the detection of CMV IgG seroconversion. However, in many pregnant women who test positive for CMV IgG during pregnancy, it remains unclear whether they were negative for CMV IgG just before the ongoing pregnancy. Therefore, tests for maternal serum CMV IgM are commonly conducted to identify primary CMV infection during ongoing pregnancy. However, universal maternal serological screening for CMV infection is not currently recommended because of several limitations. CMV IgM testing yields positive results in 4–5% of pregnant women; however, many cases arise due to persistent IgM rather than recent infections. CMV IgM might persist for 6–9 months following primary CMV infection [9] or might be detected during latent CMV reactivation [10]. Moreover, detecting cCMV in newborns from mothers who had a history of CMV infection remains challenging [11]. Traditional serology-based maternal screening is inadequate and cannot identify newborns with cCMV caused by viral reinfections or reactivations. This limitation underscores the need to shift the focus of screening from mothers to newborns.

Several newborn screening approaches have been evaluated, including dried blood spot (DBS) polymerase chain reaction (PCR)—DBS PCR—and saliva PCR. DBS PCR requires a certain viremia level at birth to be detectable; therefore, its sensitivity is inferior to that of urine or saliva testing, and it is unsuitable for screening [12]. Although saliva PCR offers the advantages of high sensitivity and ease of collection, occasional false-positive results may occur because of breast milk contamination. Therefore, confirmatory testing using a urine specimen is recommended in positive cases [13,14]. Some countries have implemented universal newborn hearing screening combined with CMV follow-up (i.e., testing newborns with failed automated auditory brainstem response (AABR)), allowing for the detection of cCMV-related sensorineural hearing loss (SNHL) that might otherwise be missed [15,16]. However, this approach alone cannot identify late-onset or progressive SNHL, and nearly half of cCMV-related SNHL cases may be missed [15]. Thus, urine-based PCR using a collection kit is a practical and highly reliable method for cCMV detection, as it overcomes the limitations of maternal serology, DBS PCR, and saliva PCR.

A prospective cohort study reported that 70% of infants with cCMV and 75% of those with symptomatic cCMV were born to mothers with nonprimary CMV infection [17]. Furthermore, a recent meta-analysis revealed comparable incidence rates of symptomatic cCMV and long-term neurological sequelae in infants born to mothers with primary and nonprimary CMV infections [18]. Antiviral agents such as valganciclovir and ganciclovir have been found to improve auditory and neurological outcomes in infants with symptomatic cCMV [19]; thus, the early diagnosis of symptomatic and asymptomatic cCMV is crucial to achieve the potential benefits of prompt intervention. Antiviral treatments might also benefit infants with asymptomatic cCMV who later develop SNHL [20].

The gold standard of cCMV diagnosis is the presence of CMV in the urine of newborns. Diagnostic tests for cCMV must be performed within the first three weeks of life because later testing does not differentiate intrauterine infection from the perinatal acquisition of CMV infection. Urine bags are commonly used to collect urine samples from newborns. However, the sample can be contaminated by stool, or body movements can cause the collection bag to detach, leading to unsuccessful collection. In girls, urine collection can be difficult because urine bags cannot always be fixed securely at the urethral meatus [21]. Dermatitis occasionally occurs when using tape fixation, and the possibility of contamination with skin bacteria increases [22]. Therefore, a urine collection method that does not involve adhesion of the urine bag to the skin is desirable. Recently, a novel urine collection kit using filter paper in newborns has been introduced. In 2019, Shino-Test Corporation (Tokyo, Japan) developed a qualitative urine collection kit containing 10 filter papers, each 3.2 mm in diameter, arranged within a urine collection sheet (International Patent Application no. PCT/JP2020/044137). This novel urine collection kit using filter papers can collect samples from neonates safely and with a high probability of success [23].

An effective screening strategy ensures early diagnosis and medical intervention to improve outcomes in infants with cCMV. This prospective cohort study aimed to evaluate the diagnostic performance of targeted screening with the use of clinical risk factors in mothers and their newborns for detecting cCMV. All newborns were assessed for cCMV using the urine collection kit with filter papers and CMV PCR tests. Newborns with positive PCR screening tests received confirmatory nucleic acid tests for cCMV diagnosis.

## 2. Materials and Methods

### 2.1. Study Design and Participants

This study was conducted to evaluate the diagnostic performance of a targeted screening approach with the use of clinical risk factors for cCMV in newborns. This prospective multicenter cohort study was conducted from October 2021 to September 2024 following the guidelines outlined in the Declaration of Helsinki. The institutional review boards of Teine Keijinkai Hospital (Approval No. 2-021231-00; approval date: 22 October 2021) and Hakodate Central General Hospital (Approval No. 2021-20; approval date: 21 October 2021) approved the study protocol. This study was also registered with the University Hospital Medical Information Network Clinical Trials Registry under registration no. UMIN000045977.

A total of 3063 pregnant women and their 3139 newborns were recruited from Hakodate Central General Hospital, Teine Keijinkai Hospital, and Miyazaki University Hospital. Written informed consent was obtained from all the women. Multiple pregnancies and pregnancies with fetal/neonatal anomalies were included, whereas pregnancies ending in miscarriage, fetal death, or stillbirth were excluded. These cases were excluded because the study focused on evaluating the diagnostic performance of targeted screening in live births, and confirmatory testing for cCMV required urine samples obtained from newborns, which could not be collected in cases of miscarriage or stillbirth.

### 2.2. Procedures

#### 2.2.1. Definition of Maternal and Neonatal Risk Factors

The following maternal and neonatal clinical findings were defined as risk factors for cCMV before participant registration: maternal fever (≥37.5 °C) or flu-like symptoms—including cough, nasal discharge, sore throat, chills, and general fatigue—during pregnancy (fever/flu-like symptoms), maternal hospitalization before 34 weeks of gestation for threatened miscarriage or preterm labor (hospitalization < 34 for TPL), preterm delivery (PD) before 34 weeks of gestation (PD < 34), ultrasound abnormalities suggesting cCMV, small for gestational age (SGA), and refer (positive) results of AABR screening (AABR refer). SGA was defined as birth weight below the 10th percentile according to the Japanese neonatal anthropometric charts for birth weight by gestational age and sex [24]. Fever/flu-like symptoms were defined as either a documented maternal body temperature ≥ 37.5 °C during pregnancy or self-reported flu-like illness (cough, nasal discharge, sore throat, chills, and general fatigue). Ultrasound abnormalities were defined as cCMV-related findings, such as microcephaly, ventriculomegaly, intracranial calcifications, pleural effusion, ascites, hepatosplenomegaly, echogenic bowel, and placentomegaly.

These maternal clinical findings were selected based on the results of our previous cohort studies, which identified the following clinical factors as risk factors for cCMV: fever/flu-like symptoms and TPL during the second trimester in primary obstetric facilities managing low-risk pregnancies [25] and fever/flu-like symptoms and fetal ultrasound abnormalities in tertiary perinatal centers managing high-risk pregnancies [26]. SGA and AABR refer, which are well known to be associated with cCMV, were also selected as neonatal clinical findings of cCMV. The Hakodate Central General Hospital, Teine Keijinkai Hospital, and Miyazaki University Hospital usually manage both low- and high-risk pregnancies. Maternal CMV IgG/IgM serological testing was not included in the study protocol because we focused on evaluating the predictive performance of a targeted screening approach based on clinical findings but not maternal antibody information.

#### 2.2.2. Screening and Diagnostic Testing

cCMV screening was performed through an outsourced testing service (Shino-Test Science Laboratories, Sagamihara, Japan). All newborns underwent CMV PCR testing of urine absorbed by filter paper within one week of birth. Urine samples were collected using a filter paper-based collection kit, which allows for simple, noninvasive sampling and is suitable for use in neonates. The urine collection kit contained 10 filter papers, each 3.2 mm in diameter, in a urine collection sheet. The filter paper was coated with a water-soluble blue dye, which turned white when water was in contact with it (International Patent Application number PCT/JP2020/044137). After urine collection, the sheet was dried and stored in a specimen box at room temperature and then mailed to Shino-Test Science Laboratories. At the laboratory, the urine sample was visually inspected for discoloration (from blue to white) and the absence of fecal contamination. Among the 10 filter papers contained in the urine collection sheet, the number of filter papers with color changed from blue to white was counted. Urine collection was considered to be successful if even one filter paper had changed color. A paper disk with a color change was selected for CMV PCR testing. No extraction or purification was performed; the disk was directly placed onto a PCR reaction plate using tweezers. The reaction mixture contained two CMV-specific primers, nucleic acid monomers (deoxyribonucleotide triphosphates), DNA polymerase, and internal control templates with dedicated primers/probes for inhibition detection. Positive and negative controls were simultaneously analyzed using filter papers dried after absorbing the CMV solution and PCR-grade water, respectively. Amplification and fluorescence detection were performed using a real-time PCR device under standard conditions. The external laboratory determined the limit of detection for this assay as 20,000 copies/mL. A comparative study between the filter paper urine PCR method and the GenelysCMV kit for definitive CMV diagnosis showed 100% concordance in an initial cohort (2/2 positive cases among 550 samples) as well as an ongoing cohort (5/5 positive cases among 898 samples) [27].

All newborns with positive PCR screening results were subjected to confirmatory nucleic acid testing using the GenelysCMV kit (Shino-Test Corporation, Tokyo, Japan) within three weeks of birth. The liquid urine sample collected using a urine collection pack was mixed with a dedicated pretreatment solution, heated at 95 °C for 90 s to release nucleic acids, and analyzed using the SmartAmp^®^ isothermal amplification method. CMV detection was based on the fluorescence generated upon the binding of Eprimer™ to the target sequence during amplification. Positive and negative results were determined based on changes in fluorescence intensity. Confirmatory testing was performed in all positive cases.

The prevalence of cCMV was determined. Newborns with cCMV underwent workups of blood tests, neurology examinations, brain imaging, AABR, ophthalmoscopy, and follow-up. They received antiviral treatments if needed.

### 2.3. Statistical Analysis

Continuous variables were presented as medians (ranges), whereas categorical variables were expressed as numbers (percentages). Newborns with and without cCMV were compared using the Mann–Whitney U test for continuous variables and Fisher’s exact test or χ^2^ test for categorical variables. A *p* value of <0.05 was considered significant. Significant predictors of cCMV were identified using a logistic regression analysis. Stepwise multivariate logistic regression analysis was conducted to eliminate confounders and determine key associations. Criteria of *p* < 0.10 for entry and *p* ≥ 0.05 for removal were used in this analysis. The odds ratios (ORs) and their 95% confidence intervals (CIs) were calculated.

Only nine cases of cCMV were identified in our cohort, so the events-per-variable ratio was <10, raising concerns regarding potential overfitting. To minimize this risk, we performed univariate logistic regression for each of the six predefined clinical risk factors individually, rather than constructing a multivariable model including all predictors. No penalized regression techniques were applied because the number of candidate variables was limited and our primary aim was exploratory. Multicollinearity was not assessed because each variable was analyzed separately, and no data were missing for any of the predefined clinical risk factors, as variables were prospectively collected for all participants.

The diagnostic performance of maternal and neonatal risk factors in predicting cCMV was assessed by computing the sensitivity, specificity, positive predictive value (PPV), negative predictive value (NPV), and overall accuracy. The Youden index (sensitivity + specificity − 1) was utilized to evaluate the discriminatory ability of each risk factor. Statistical analyses were conducted using EZR (Easy R) version 1.68 [28].

## 3. Results

### 3.1. Study Cohort and Incidence of cCMV

**Figure 1:**
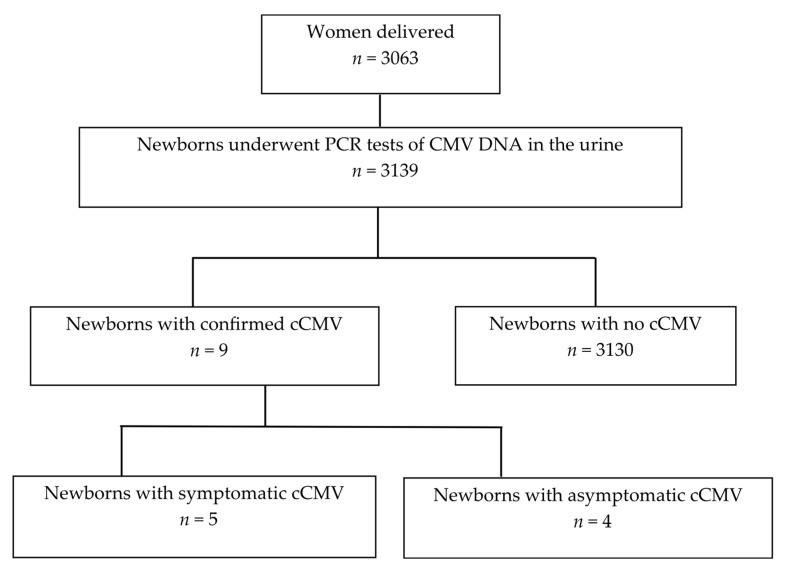
Flowchart of the study cohort. CMV, cytomegalovirus; cCMV, congenital cytomegalovirus infection.

Figure 1 shows a flowchart of the study cohort. Between October 2021 and September 2024, a total of 3139 newborns, comprising 2986 singletons, 144 twins, and nine triplets, born to 3063 mothers, were subjected to urine PCR testing for CMV DNA. Nine newborns tested positive for CMV PCR testing in urine as well as confirmatory nucleic acid testing for cCMV diagnosis, yielding a cCMV incidence of 0.29% (one in three hundred and forty-nine newborns). The remaining 3130 newborns tested negative for CMV PCR testing in urine absorbed by filter paper. Five and four newborns were diagnosed with symptomatic and asymptomatic cCMV, respectively.

### 3.2. Clinical Background and Frequency of the Six Risk Factors for cCMV

Table 1 summarizes the clinical background and the frequency of six predefined risk factors for cCMV in our cohort. Mothers of infants with cCMV (median age, 28 years; range, 18–39 years) were younger than those of non-cCMV (median age, 32 years; range, 15–51 years; *p* = 0.021). However, there were no significant differences in gestational weeks at delivery, body mass index, rates of primipara, multiple pregnancies, or PD before 37 weeks of gestation (PD < 37) between the two groups. The clinical backgrounds of mothers with multiple pregnancies were analyzed repeatedly according to the number of newborns. Among the six risk factors, fever or flu-like symptoms (33.3% vs. 6.2%; *p* = 0.0156), fetal ultrasound abnormalities (33.3% vs. 2.7%; *p* = 0.0016), SGA (44.4% vs. 10.5%; *p* = 0.0037), and AABR refer (55.6% vs. 1.6%; *p* < 0.0001) were significantly more prevalent in newborns with cCMV than in those without cCMV. Hospitalization < 34 for TPL and PD < 34 were not significantly different between the two groups.

### 3.3. Nine Cases with cCMV

Table 2 shows the clinical findings, including the six predefined risk factors of nine cases with cCMV. All nine cases were positive for at least one of the six risk factors. Three cases (Cases #3, #5, and #6) had fever/flu-like symptoms; two (Cases #2 and #7) had hospitalization < 34 for TPL; three (Cases #1, #2, and #9) had fetal ultrasound abnormalities; one (Case #7) had PD < 34; four (Cases #2, #4, #7, and #9) had SGA; and five (Cases #1, #2, #5, #8, and #9) had AABR refer. Six of the nine cCMV-positive cases had two or more overlapping risk factors, whereas three cases had only a single risk factor (Cases #6, #7, and #5 with maternal fever/flu-like symptoms, SGA, and AABR refer, respectively).

Five infants (Cases #1, #2, #4, #5, and #9) were classified as having symptomatic cCMV, while the remaining four infants (Cases #3, #6, #7, and #8) were classified as asymptomatic. In this study, symptomatic cCMV was defined as the presence of abnormal clinical, imaging, or hearing findings identified at birth or during immediate neonatal workup, such as ventriculomegaly, hepatosplenomegaly, abnormal AABR, or brain magnetic resonance imaging (MRI) abnormalities. Infants without such findings were classified as asymptomatic cCMV. For example, Case #7 had only SGA and was classified as asymptomatic. Four infants (Cases #1, #4, #5, and #9) received valganciclovir therapy. Three infants developed sequelae, including hearing loss (Cases #1 and #9) and cerebral palsy (Case #2), whereas the remaining six infants developed normally without sequelae.

This targeted screening approach could detect six newborns with cCMV based on the presence of clinical risk factors other than fetal ultrasound abnormalities (Cases #3, #4, #5, #6, #7, and #8). The abnormality of white matter of the brain was found by MRI in Case #4. Hearing abnormality was found by AABR tests in Case #5. These two infants were found to have mild symptoms detected by workups performed after cCMV diagnosis and subsequently received valganciclovir therapies, having normal development without sequelae.

### 3.4. Risk Factors for cCMV and Logistic Regression Analysis

Table 3 shows the logistic regression analysis of the six risk factors for cCMV. A total of 925 (29.5%) of 3139 newborns had at least one of the six clinical risk factors for cCMV. In the univariate logistic regression analysis, fever or flu-like symptoms (odds ratio (OR), 7.5; 95% CI, 1.9–30.3), fetal ultrasound abnormalities (OR, 28.7; 95% CI, 7.6–108.6), SGA (OR, 6.8; 95% CI, 1.8–25.6), and AABR refer (OR, 75.5; 95% CI, 19.7–289.2) were found to be significant risk factors. The frequencies of hospitalization < 34 for TPL (OR, 2.4; 95% CI, 0.50–11.6) and PD < 34 (OR, 2.2; 95% CI, 0.27–17.6) in newborns with cCMV were approximately two-fold higher than in those with non-cCMV; however, the difference was not statistically significant. In the multivariate stepwise regression analysis, fever/flu-like symptoms (OR, 12.3; 95% CI, 2.4–63.3), fetal ultrasound abnormalities (OR, 11.9; 95% CI, 3.2–100.8), and AABR refer (OR, 62.2; 95% CI, 13.2–292.8) remained as independent risk factors for cCMV. A total of 597 (19.0%) newborns had at least one of the four significant risk factors (fever/flu-like symptoms, fetal ultrasound abnormalities, SGA, and AABR refer).

### 3.5. Diagnostic Performance of Targeted Screening

The diagnostic performance of each risk factor for cCMV, including sensitivity, specificity, PPV, NPV, accuracy, and Youden index, is shown in Table 4. The sensitivity of AABR refer and SGA was relatively high, while the PPV of AABR refer and fetal ultrasound abnormalities was relatively high.

When at least one of the six risk factors (fever/flu-like symptoms, hospitalization < 34 for TPL, PD < 34, fetal ultrasound abnormalities, SGA, and AABR refer) was present, the diagnostic performance yielded 100% sensitivity (95% CI, 55.5–100), 70.7% specificity (95% CI, 69.1–72.3), 1.0% PPV (95% CI, 0.4–1.8), 100% NPV (95% CI, 99.7–100), and an overall accuracy of 70.8% (95% CI, 69.2–72.4) with a Youden index of 0.707.

When at least one of the four significant risk factors (fever/flu-like symptoms, fetal ultrasound abnormalities, SGA, and AABR refer) identified by the logistic analyses was present, 100% sensitivity (95% CI, 55.5–100), 81.2% specificity (95% CI, 79.8–82.6), PPV of 1.5% (95% CI, 0.7–2.8), NPV of 100% (95% CI, 99.8–100), and overall accuracy of 81.3% (95% CI, 79.9–82.6) with a maximum Youden index of 0.812 were achieved (Table 4). Thus, the targeted screening with the use of clinical risk factors in mothers and their newborns effectively identified cCMV.

## 4. Discussion

This cohort study defined maternal and neonatal clinical findings—fever/flu-like symptoms, hospitalization < 34 for TPL, PD < 34, fetal ultrasound abnormalities, SGA, and AABR refer—as risk factors for cCMV before participant registration. A total of 925 (29.5%) of 3139 newborns had at least one of the six risk factors. The results demonstrated that this targeted screening with the use of the six predefined risk factors achieved 100% sensitivity and 70.7% specificity for detecting cCMV in newborns. In contrast, logistic regression identified four factors—fever/flu-like symptoms, fetal ultrasound abnormalities, SGA, and AABR refer—as statistically significant predictors of cCMV. Targeted screening with the use of the four predictors yielded 100% sensitivity while improving the specificity to 81.2% with a maximum Youden index for detecting cCMV. Importantly, this refinement indicated that only 597 (19.0%) newborns were required to undergo urine nucleic acid testing, without compromising case detection.

Universal neonatal screening by urinary nucleic acid testing is the gold standard for identifying newborns with cCMV; however, financial and logistical constraints limit its widespread implementation. Nevertheless, targeted screening using these risk factors might be a viable alternative to universal neonatal screening, offering a highly sensitive yet cost-effective method for the early detection of cCMV. When this targeted screening is implemented, only 19.0–29.5% of newborns who had these four to six risk factors for cCMV are supposed to undergo urine nucleic acid tests for cCMV diagnosis.

This study revealed relatively high sensitivity of AABR refer and SGA and relatively high PPV of AABR refer and fetal ultrasound abnormalities for cCMV diagnosis. At present, neonatal testing for cCMV is primarily performed when fetal ultrasound abnormalities indicative of CMV infections are detected or when newborns have AABR refer. However, cCMV has a broad clinical spectrum, with varying severity of symptoms. Although microcephaly has been identified as a strong predictor of cCMV [29,30], many other clinical manifestations are not specific, making it challenging to determine which cases warrant confirmatory testing.

cCMV is a major cause of pediatric hearing impairment. It is the second most common cause of childhood hearing loss after genetic etiologies, accounting for 10–20% of all childhood hearing loss cases [31] and 15–20% of moderate-to-severe hearing loss cases [32]. To identify infants at risk for SNHL earlier, targeted CMV screening of newborns who are referred for hearing screening has been performed in the USA [16,33]. This approach identifies infants with asymptomatic cCMV and cCMV-related SNHL that would have otherwise not been detected. Another study reported the added benefit of this approach in improving the 3-month diagnostic hearing evaluation rate for the newborn hearing screening program [26]. However, a recent large multicenter study found that it failed to detect late-onset hearing loss, and the targeted CMV hearing screening approach would miss 43% of infants with CMV-related SNHL [15]. Similarly, this present study demonstrated that the sensitivity of AABR refer for detecting cCMV in the neonatal period was 55.6% (5/9), and the targeted hearing screening would miss the remaining 44.4% of newborns with cCMV.

Given that most newborns with cCMV are asymptomatic at birth, it is not easy to identify cCMV in the newborn period. Universal screening for cCMV in all newborns assures early diagnosis and medical intervention to improve outcomes for infants with SNHL, with a recent study showing the cost-effectiveness of such an approach [34]. Diagnostic tests for cCMV must be performed within the first three weeks of life because later testing cannot differentiate intrauterine infection from perinatal acquisition of CMV infection. However, DBS PCR tests have shown highly varied sensitivity while screening newborns for cCMV [35,36,37]. Because high viral loads are shed in both the urine and saliva of newborns with cCMV, both specimens are reliable for cCMV diagnosis [38,39,40]. Saliva CMV PCR has also been used for cCMV screening [41]. Although CMV present in breast milk could lead to false-positive results when using saliva PCR for CMV screening in newborns, a large study demonstrated an acceptably low false-positive rate [42]. Obtaining a saliva sample at least 1 h after breastfeeding could avoid potential contamination with CMV from breast milk. The cCMV can be diagnosed when CMV is present in the urine of newborns within three weeks of life. This present study applied a qualitative urine collection kit containing filter papers and CMV PCR tests for screening in all newborns and performed confirmatory urine nucleic acid tests for the diagnosis of cCMV in the nine newborns with positive PCR screening tests.

Several observational studies have reported varying incidences of cCMV depending on maternal and neonatal clinical findings: 1.1–1.4% in pregnancies with maternal fever or flu-like symptoms, 61.1% with fetal ultrasound abnormalities, 0.8–2% with TPL, 1.2–1.3% with PD, 0.8–3.7% with SGA, 2.9–3.3% with multiple pregnancy, and 5.0% in newborns with AABR refer [8,43,44,45,46,47]. These findings suggest that targeted screening based on these clinical factors for detecting cCMV may be more effective than universal maternal CMV antibody screening, where the incidence of cCMV is approximately 1% among women who tested positive for both CMV IgG and CMV IgM during pregnancy [8,47].

Recent prospective cohort studies have assessed maternal clinical predictors in different obstetric settings. Fever/flu-like symptoms (OR 19.8; 95% CI, 4.1–95.7) and second-trimester TPL (OR 7.1; 95% CI, 1.9–26.7) have been identified as significant risk factors of cCMV in primary obstetric facilities managing low-risk pregnancies [25]. In contrast, young maternal age (<25 years; OR 2.7; 95% CI, 1.1–6.6), fever/flu-like symptoms (OR 5.4; 95% CI, 2.6–11.2), fetal ultrasound abnormalities (OR 12.7; 95% CI, 5.8–27.7), and PD < 34 weeks (OR 2.6; 95% CI, 1.1–6.0) have been highlighted as key predictors of cCMV in tertiary perinatal centers managing high-risk pregnancies [26].

Thus, in this present cohort study, these maternal clinical findings (fever/flu-like symptoms, hospitalization < 34 for TPL, PD < 34, and fetal ultrasound abnormalities) together with neonatal clinical findings of SGA and AABR refer, which are well known to be associated with cCMV, were evaluated as potential predictors of cCMV. This targeted screening approach successfully identified all newborns with cCMV in our cohort. Importantly, this method can be employed during routine pregnancy checkups in various clinical settings, potentially reducing cCMV-related morbidity and improving long-term outcomes for affected infants.

However, the confidence intervals for sensitivity (100%, 95% CI 55.5–100) and specificity (70.7%, 95% CI 69.1–72.3) in this study were wide, reflecting the limited number of cCMV-positive cases (*n* = 9). Given this small sample size, the sensitivity estimate is statistically fragile, as missing even a single case would substantially lower the calculated value. Therefore, the reliability of the performance estimates is limited, and larger, multicenter validation studies are warranted to confirm the utility and generalizability of this targeted screening approach.

In our cohort, the incidence of cCMV was 0.29% (9/3139), which was lower than the 0.5–2% generally reported in developed countries. However, this rate is not unusually low for Japan. Previous studies have reported similar incidences, such as 0.31% [48] and 0.22% [25], suggesting that the prevalence observed in this present study reflects the baseline epidemiology in Japanese populations rather than underdiagnosis or a methodological limitation.

Public awareness of cCMV remains low among women of childbearing age. Education on CMV infection—ideally initiated before pregnancy—is crucial for reducing maternal infection risk through appropriate hygiene practices [49,50,51]. Currently, a combination of universal public education on CMV prevention for all pregnant women, regardless of their CMV antibody status, and targeted newborn urine screening can be an effective strategy for reducing the burden of cCMV. Future studies should focus on validating the findings of this study across different populations and refining the screening criteria to further optimize detection rates of cCMV.

## 5. Conclusions

This cohort study revealed maternal and neonatal clinical findings—fever/flu-like symptoms, hospitalization < 34 for TPL, PD < 34, fetal ultrasound abnormalities, SGA, and AABR refer—as risk factors for cCMV. A total of 925 (29.5%) of 3139 newborns had at least one of these six risk factors. The targeted screening approach with the use of these six risk factors yielded 100% sensitivity and 70.7% specificity for detecting cCMV in newborns. Meanwhile, a targeted screening with the use of four risk factors determined by logistic regression analyses—fever/flu-like symptoms, fetal ultrasound abnormalities, SGA, and AABR refer—yielded 100% sensitivity and 81.2% specificity, with the maximum Youden index for cCMV detection. Only 597 (19.0%) newborns had at least one of the four risk factors. The proposed targeted screening method demonstrated high effectiveness in identifying newborns with cCMV, achieving 100% sensitivity. When this targeted screening is employed, 19.0–29.5% of the newborns—those presenting with at least one of the four to six clinical risk factors—would require urine nucleic acid testing for cCMV diagnosis. Considering the infrastructural and financial limitations in healthcare settings, this targeted screening may serve as a feasible and cost-effective alternative to universal urine screening for cCMV.

## Figures and Tables

**Table 1:** Clinical background and frequency of six risk factors for congenital cytomegalovirus infection.

	Total *n* = 3139	cCMV *n* = 9	Non-cCMV *n* = 3130	*p* Value
Clinical background				
Maternal age, years	32 (15–51)	28 (18–39)	32 (15–51)	0.021
Gestational weeks at delivery	38 (22–42)	38 (33–40)	38 (22–42)	0.419
Primipara	1524 (48.6%)	4 (44.4%)	1520 (48.6%)	1
Multiple pregnancy	153 (4.9%)	0 (0%)	153 (4.9%)	1
Body mass index, kg/m^2^	21.8 (13.6–45.7)	21.7 (17.1–44.6)	21.8 (13.6–45.7)	0.793
PD < 37 weeks	392 (12.5%)	2 (22.2%)	390 (12.5%)	0.312
Risk factors for cCMV				
Fever/flu-like symptoms	198 (6.3%)	3 (33.3%)	195 (6.2%)	0.0156
Hospitalization < 34 for TPL	335 (10.7%)	2 (22.2%)	333 (10.6%)	0.248
PD < 34	170 (5.4%)	1 (11.1%)	169 (5.4%)	0.395
Fetal ultrasound abnormalities	88 (%)	3 (33.3%)	85 (2.7%)	0.0016
SGA	332 (2.8%)	4 (44.4%)	328 (10.5%)	0.0037
AABR refer	56 (1.8%)	5 (55.6%)	51 (1.6%)	<0.0001

Median (range). cCMV, congenital cytomegalovirus infection; PD, preterm delivery; hospitalization < 34 for TPL, hospitalization for threatened miscarriage or preterm labor before 34 weeks of gestation; PD < 34, preterm delivery before 34 weeks of gestation; SGA, small for gestational age; AABR, automated auditory brainstem response.

**Table 2:** Clinical findings of nine cases with congenital cytomegalovirus infection.

Case	Age (Year)	Gravidity/Parity	BMI Before Pregnancy (kg/m^2^)	Gestational Week at Delivery	Birth Weight (g)	Sex	Fever/ Flu-like Symptoms (GW)	Hospitalization < 34 for TPL (GW)	Fetal Ultrasound Abnormalities (GW)	PD < 34 (GW)	SGA	AABR Refer	Symptoms of Newborns with cCMV	Antiviral Therapy	Development/Sequelae (Age)
1	18	1/0	24.2	37	2674	Male	No	No	Ventriculomegaly (37)	No	No	Presence	Ventriculomegaly, hearing abnormality	Valganciclovir	Hearing loss (3 y and 4 mo)
2	29	1/0	17.1	39	2291	Male	No	Presence (24–26)	Ventriculomegaly (39)	No	Presence	Presence	SGA, hypoxic-ischemic encephalopathy, bilateral internal carotid artery occlusion	No	Cerebral palsy (3 y and 1 mo)
3	31	3/2	24.2	38	3252	Female	Presence (12)	No	No	No	No	No	Asymptomatic	No	Normal (2 y and 10 mo)
4	28	3/1	20.2	38	2274	Male	No	No	No	No	Presence	No	SGA, white matter abnormality of the brain (MRI)	Valganciclovir	Normal (2 y and 8 mo)
5	25	2/1	20.3	35	2362	Male	Presence (32, 35)	No	No	No	No	Presence	Hearing abnormality	Valganciclovir	Normal (2 y and 6 mo)
6	23	3/1	28.4	40	3580	Female	Presence (13)	No	No	No	No	No	Asymptomatic	No	Normal (1 y and 0 mo)
7	39	2/0	21.7	33	1657	Female	No	Presence (31–33)	No	Presence (33)	Present	No	SGA, asymptomatic	No	Normal (2 y and 2 mo)
8	25	1/0	44.6	40	2920	Female	No	No	No	No	No	Presence	Asymptomatic	No	Normal (1 y and 0 mo)
9	31	5/2	19.8	37	1702	Male	No	No	Ventriculomegaly, intracranial calcifications, hepatosplenomegaly, echogenic bowel (23)	No	Presence	Presence	SGA, ventriculomegaly, hearing abnormality	Valganciclovir	Hearing loss (11 mo)

BMI, body mass index; GW, gestational week; hospitalization < 34 for TPL, hospitalization for threatened miscarriage or preterm labor before 34 weeks of gestation; PD < 34, preterm delivery before 34 weeks of gestation; SGA, small for gestational age; AABR, automated auditory brainstem response; MRI, magnetic resonance imaging.

**Table 3:** Logistic regression analysis of six risk factors for congenital cytomegalovirus infection.

			Univariate Analysis			Forward Stepwise Multivariate Regression		
Risk Factors for cCMV	cCMV *n* = 9	Non-cCMV *n* = 3130	OR	95% CI	*p* Value	OR	95% CI	*p* Value
Fever/flu-like symptoms	3 (33.3%)	195 (6.2%)	7.5	1.9–30.3	0.0045	12.3	2.4–63.3	0.0026
Hospitalization < 34 for TPL	2 (22.2%)	333 (10.6%)	2.4	0.50–11.6	0.28			
PD < 34	1 (11.1%)	169 (5.4%)	2.2	0.27–17.6	0.46			
Fetal ultrasound abnormalities	3 (33.3%)	85 (2.7%)	28.7	7.6–108.6	<0.0001	11.9	3.2–100.8	0.0011
SGA	4 (44.4%)	328 (10.5%)	6.8	1.8–25.6	0.0043	4.2	0.7–24.3	0.1087
AABR refer	5 (55.6%)	51 (1.6%)	75.5	19.7–289.2	<0.0001	62.2	13.2–292.8	<0.0001

cCMV, congenital cytomegalovirus infection; OR, odds ratio; CI, confidence interval; hospitalization < 34 for TPL, hospitalization for threatened miscarriage or preterm labor before 34 weeks of gestation; PD < 34, preterm delivery before 34 weeks of gestation; SGA, small for gestational age; AABR, automated auditory brainstem response.

**Table 4:** Diagnostic performance of risk factors for congenital cytomegalovirus infection.

Risk Factors for cCMV	Sensitivity (95% CI)	Specificity (95% CI)	Positive Predictive Value (95% CI)	Negative Predictive Value (95% CI)	Accuracy % (95% CI)	Youden Index
Fever/flu-like symptoms	0.333 (0.075–0.701)	0.938 (0.929–0.946)	0.015 (0.003–0.044)	0.998 (0.996–0.999)	0.936 (0.927–0.944)	0.271
Hospitalization < 34 for TPL	0.222 (0.028–0.600)	0.894 (0.882–0.904)	0.006 (0.001–0.021)	0.998 (0.995–0.999)	0.892 (0.880–0.902)	0.116
PD < 34	0.111 (0.003–0.482)	0.946 (0.938–0.954)	0.006 (0.000–0.032)	0.997 (0.995–0.999)	0.944 (0.935–0.951)	0.057
Fetal ultrasound abnormalities	0.333 (0.075–0.701)	0.974 (0.967–0.979)	0.034 (0.007–0.096)	0.998 (0.996–0.999)	0.972 (0.965–0.977)	0.307
SGA	0.444 (0.137–0.788)	0.895 (0.884–0.906)	0.012 (0.003–0.031)	0.998 (0.996–0.999)	0.894 (0.883–0.904)	0.339
AABR refer	0.556 (0.212–0.863)	0.984 (0.979–0.988)	0.089 (0.030–0.196)	0.999 (0.997–1.000)	0.982 (0.977–0.987)	0.54
Any of the above six risks	1.000 (0.555–1.000)	0.707 (0.691–0.723)	0.010 (0.004–0.018)	1.000 (0.997–1.000)	0.708 (0.692–0.724)	0.707
Any of the four risks (fever/flu-like symptoms, fetal ultrasound abnormalities, SGA, AABR refer)	1.000 (0.555–1.000)	0.812 (0.798–0.826)	0.015 (0.007–0.028)	1.000 (0.998–1.000)	0.813 (0.799–0.826)	0.812

cCMV, congenital cytomegalovirus infection; CI, confidence interval; hospitalization < 34 for TPL, hospitalization for threatened miscarriage or preterm labor before 34 weeks of gestation; PD < 34, preterm delivery before 34 weeks of gestation; SGA, small for gestational age; AABR, automated auditory brainstem response.

## Data Availability

The data presented in this study are available on request from the corresponding author due to ethical and privacy restrictions.

## Abbreviations

The following abbreviations are used in this manuscript:

CMV	cytomegalovirus
cCMV	congenital cytomegalovirus infection
AABR	automated auditory brainstem response
PD	preterm delivery
SNHL	sensorineural hearing loss
TPL	threatened miscarriage or preterm labor
